# Detection of Epstein–Barr virus infection in thymic epithelial tumors by nested PCR and Epstein–Barr-encoded RNA ISH

**DOI:** 10.1186/s13027-023-00497-9

**Published:** 2023-06-09

**Authors:** Li Zhao, Jian-Yong Ding, Yun-Lan Tao, Kun Zhu, Gang Chen

**Affiliations:** 1 Department of Pathology, Zhongshan Hospital, Fudan University, Shanghai, China; 2 Department of Thoracic Surgery, Zhongshan Hospital, Fudan University, Shanghai, China

**Keywords:** EBV, Thymic epithelial tumors, Nested PCR, EBER

## Abstract

**Background:**

Epstein–Barr virus (EBV) is well known to be associated with a lot of tumors, including lymphoma, nasopharyngeal carcinoma, EBV-associated gastric carcinoma, and some other carcinomas with similar lymphoepithelioma-like features. However, the association between EBV and thymic epithelial tumors (TETs) is inconclusive as reports in this regard are not entirely consistent and the methods employed are of different sensitivity and specificity. The geographical difference of the patients is also one of the reasons for the different points of view.

**Methods:**

In our study, we examined 72 thymomas, including 3 cases of type A thymomas, 27 cases of type AB, 6 cases of type B1, 26 cases of type B2 and 10 cases of type B3 thymomas, and 15 thymic carcinomas to detect the viral genome at both DNA and RNA levels. The genome DNA of fresh tissues was first screened by nested polymerase chain reaction (PCR), which could be regarded as the most sensitive method to detect small amounts of DNA. Then all the tissue blocks were further submitted for viral localization by Epstein–Barr-encoded RNA (EBER) ISH. Group parameters were assessed using the chi-square test at a significance level of p < 0.05.

**Results:**

Nested PCR results showed that none of type A, eight (29.6%) type AB, one (16.7%) type B1, fifteen (57.7%) type B2, and four (40.0%) type B3 were positive for EBV genome. However, none of them detected EBER expression except for one case of type B2 thymoma. Fourteen (93.3%) thymic carcinomas were positive for EBV by nested PCR, of which three displayed weak nuclear signals within the tumor cells by EBER ISH.

**Conclusions:**

These results showed that nested PCR was a sensitive method for screening the EBV genome in thymic epithelial tumors. As the malignancy of thymoma increases, the rate of EBV infection became higher. Thymic carcinomas were well associated with the Epstein–Barr virus.There was significant association between the EBV infection rate and thymoma type (p < 0.05). We further analyzed the association between EBV infection and myasthenia gravis. However, it showed no significant difference(p = 0.2754), although the EBV infection rate was higher in the thymomas with myasthenia gravis.

## Introduction

Epstein–Barr virus was firstly introduced in 1964 from a Burkitt’s lymphoma patient’s (Burkett’s lymphoma, BL)living tissue and was separated during inspection [1]. It is a ubiquitous cause of infection in the human world widely. 2013 American survey found that the sero-positive rate of children aged 6 to 8 years was 50%, 18- to 19-year-old positive rate was 89% [2]. Epstein-Barr virus initially enters the human body by infecting human oral epithelial cells, and then invades the human B lymphocytes, and can be latently infected in the human body for a lifetime. The EBV genome is approximately 172 kb and consists of a linear double-stranded DNA molecule that can encode more than 85 genes. The coding genes are currently known as 6 nuclear antigens (EBV nuclear antigen, EBNA), 3 latent membrane proteins (LMP), small non-polyadenylated RNAs, EBER1 and 2, microRNA and several early lytic genes [3].

The role of EBV is well known in the pathogenesis of infectious mononucleosis, Burkitt’s lymphoma, and nasopharyngeal carcinoma [4, 5]. EBV has also been detected in a variety of non-nasopharyngeal carcinomas, including carcinomas of the salivary glands, lungs and stomach, mostly with similar lymphoepithelioma-like features [6–8].

Thymic epithelial tumors (TETs) are rare thymic neoplasms including a series of tumors characterized by lymphoepithelioma-like features. Although the correlation between TETs and EBV infection has been reported previously. However, the association between EBV infection and thymic epithelial tumors is controversial.

TETs include two broad categories: thymoma and thymic carcinoma. Thymoma is defined as an organotypic tumor derived from thymic epithelium, with low-grade cytology. According to the fifth edition of the WHO classification of thymic epithelial tumors, there are five different types of thymic tumors (A, AB, B1, B2, and B3) [9]. Based on the shape of the neoplastic cell: type A and type B thymomas are described. Type A thymomas have the best prognosis and are usually low stage. Type AB tumors are similar to type A, but have foci of neoplastic lymphocytes. Type B thymomas are sub-divided into three categories (B1, B2, and B3) on the basis of the proportional increase in thymocytes and cellular atypia. As lesions progress from A to B3, there is also a progressive deterioration of the prognosis. Thymic carcinoma is a heterogeneous group of high-grade malignant thymic epithelial neoplasms. All Western and Asian series failed to demonstrate the roles EBV played in thymomas. McGuire et al. from Hong Kong reported two thymomas and three of five thymic lymphoid hyperplasia were positive for the EBV genome [10]. Chen PC examined EBV of 78 thymomas and 21 thymic carcinomas in Taiwanese patients at both DNA and RNA levels, they found none of the thymomas showed a detectable EBV genome. Eight thymic carcinomas were positive for EBV by nested PCR, of which six displayed nuclear signals within the tumor cells by in situ PCR ISH and/or RNA ISH [11]. Due to regional differences in EBV infection as well as TETs rare incidence, investigators could not have a unified cognition. Since the EBV infection rate increased in recent years, this prompted us to undertake the present investigation to address the issue more thoroughly, we speculated that there might be a higher likelihood of EBV involvement in thymic epithelial tumors.

## Methods

### Case collection

72 cases of thymoma and 15 cases of thymic carcinomas were retrieved from the Zhongshan Hospital Affiliated with Fudan University. The pathological slides were reviewed and the tumors were classified according to the newly published WHO classification in 2015 [12]. The thymomas included 3 cases of type A thymomas, 27 cases of type AB thymomas, 6 cases of type B1 thymomas, 26 cases of type B2 thymomas, and 10 cases of type B3 thymomas. The patients’ ages ranged from 18 years to 74 years old, with a median of 54 years old in the thymoma group, and from 44 years to 76 years, with a median of 58 years in the thymic carcinoma group. 9 cases of thymomas and 2 cases of thymic carcinomas had MG, respectively.

### DNA extraction

Firstly, grind the tissue and add 460 µl nuclear lysate into a 1.5 ml EP tube, add 20 µl 20 mg/ml proteinase K (20 mg/ml) and 20 µl 10% SDS, 58℃ overnight, the next day add 5 µl of RNase for 2–4 h. Then add 500 µl phenol-chloroform (PH > 7.8) into the EP tube, after centrifugation (13.0 × 10^3^ g) for 20 min, it is divided into 3 layers, the upper layer is the DNA layer, suck the upper layer, add an equal volume of isopropanol and centrifuge for seconds. Add 1/10 volume of 3 M NaAC of the upper aspirate liquid, 4℃ 12,000 rpm 35 min, wash twice with 900 µl 75% RNA-free alcohol, and finally add RNase-free water to dissolve DNA.

### Nested PCR amplification

The first and second PCR conditions were both 35 cycles at 94℃, 30 seconds, 55℃, 30 seconds, and 72℃, 30 seconds. The first PCR amplification was performed using an external pair of primers with an upstream sequence of 5’-TTCATCACCGTCGCTGACT-3’ and a downstream sequence of 5’-ACCGCTTACCACCTCCTCT-3’. The first round PCR amplified a 298-base pair (bp) DNA fragment. The second PCR amplification was then performed using 1 µl of the first PCR product as a template and an internal pair of primers with an upstream sequence of 5’-CCAGAGGTAAGTGGACTT-3’ and a downstream sequence of 5’-GACCGGTGCCTTCTTAGG-3’. These two primer sets amplified a 122 bp DNA fragment within the first tandem internal repeats of EBV. After electrophoresis and gene green staining, the PCR product was visualized in a UV box. The amplified DNA product had been verified by first-generation sequencing.

### EBER RNA ISH

4 μm sections of formalin-fixed, paraffin-embedded tissue blocks were mounted on silicon-coated glass slides, dried on a 56℃ oven overnight, deparaffinized, rehydrated, and digested by proteinase K (20 mg/ml), following the standard protocol. ISH was performed using a dig-labeled EBER riboprobe. Hematoxylin was used as a counterstain.

### Statistic

The association of EBV infection in different groups were assessed using Chi-square test. A p-value < 0.05 was statistically significant.

## Results

### TETs showed a detectable EBV genome by nested PCR

**Fig. 1:**
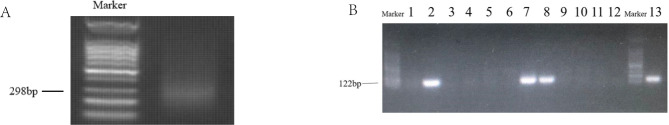
Genome DNA nested PCR gel electrophoresis. Detection of EBV DNA by nested PCR after the first round of amplification. The 298 bp product was seen in lane (A). One of the representative results was shown here. And the 122 bp amplification products were seen in lanes 2, 7 and 8 after the second round of amplification. No bands were visualized in lanes 3, 4, 5, 6, 9, 10, 11 and 12. Lane 1 and 13 were the negative and positive control, respectively (B). The lane marked “Marker” is the 100 bp ladder (100–1500 bp) DNA marker

Nested PCR is a sensitive method to test the EBV genome. We detected 72 thymomas and 15 thymic carcinomas for the EBV infection. The nested PCR results showed that none of the type A thymoma was positive for the EBV genome. Eight of twenty-seven (29.6%) cases of type AB thymomas were positive for the EBV genome. One (16.7%) type B1, fifteen (57.7%) type B2, and four (40.0%) type B3 thymomas were detected to have EBV infection, respectively. 14 of 15 thymic carcinomas were EBV positive.Chi-square test showed a significant association between the EBV infection rate and thymoma type (p < 0.05) Table 1. And we can see a 298 bp amplification product of PCR first and then a final 122 bp amplification product (Fig. 1A, B). The final amplification product was also checked by first-generation sequencing.

**Table 1:** The EBV positive rate in different groups

TETs Type	EBV Negative	EBV Positive	p	Chi-square
A	3	0	0.0001048 18.762	
AB	19	8		
B	22	20		
SCC	1	14		

Chi-square test

### One case of B2 thymoma and three thymic carcinomas were positive for EBER

**Fig. 2:**
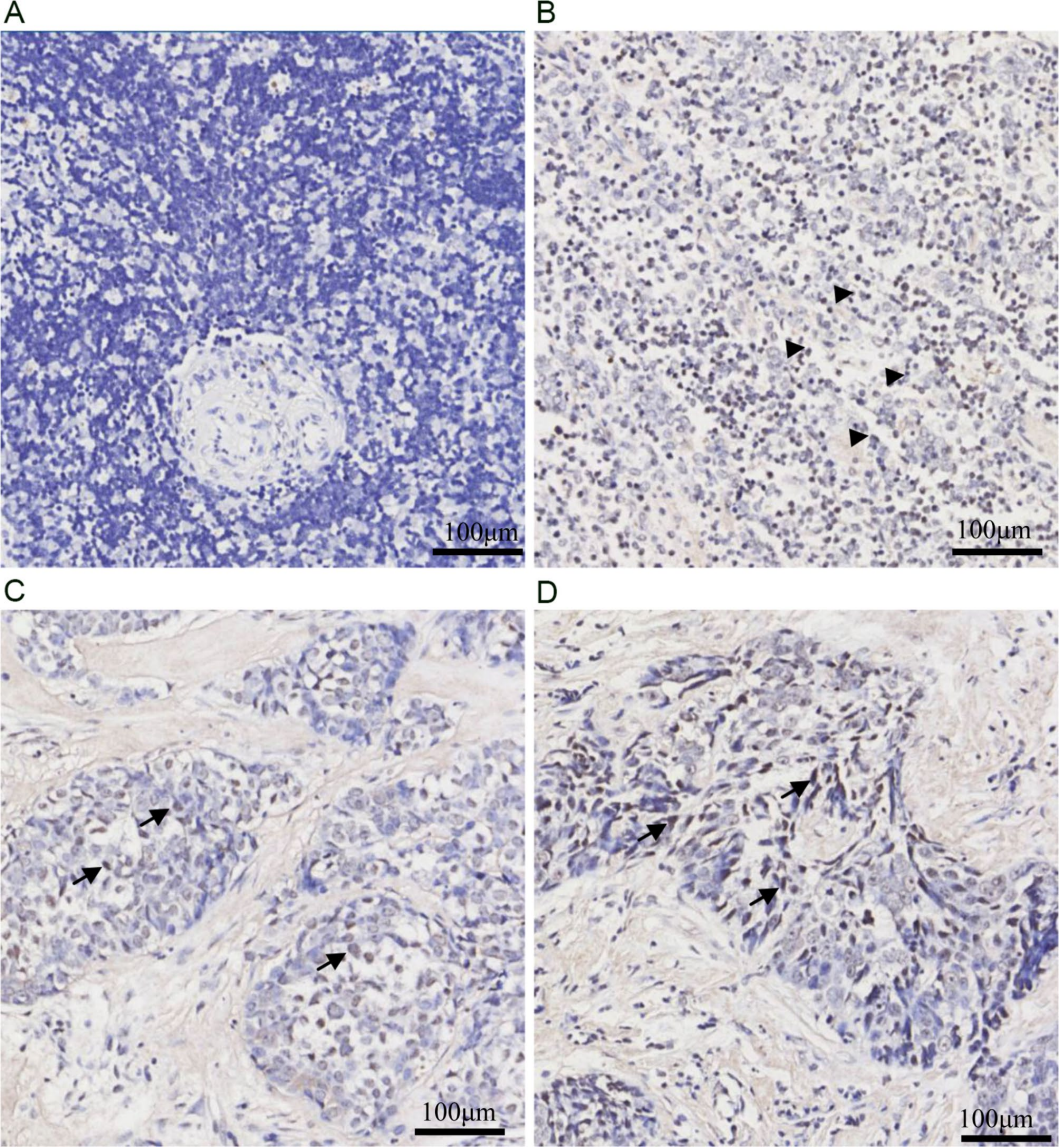
One case of type B2 thymoma and 2 cases of thymic carcinomas were positive for EBER. (A) No signals were visible either in lymphocytes or in tumor cells. (B) The B2 thymoma showed positive signals only within the infiltrating lymphocytes (arrow heads). (C, D) EBER ISH showed nuclear signals within the tumor cells of thymic carcinoma (arrow, x100)

All of the above cases were also verified by EBER RNA ISH. However, most cases were negative for EBER (Fig. 2A). Only one type B2 thymoma showed positive signals within the infiltrating lymphocytes (Fig. 2B). Three thymic carcinomas, which were positive in nested PCR, showed positive nuclear signals within the tumor cells by EBER RNA ISH (Fig. 2C, D).

### EBV was related to thymoma-associated myasthenia gravis

The information of EBV infection and myasthenia gravis (MG) of patients was shown in Table 2. In the MG + group, the EBV positive rate was 63.6% (7/11), which was higher than the MG- group 43.4% (33/76). It showed no significant difference between EBV infection and thymoma-associated myasthenia gravis (p = 0.2754) Table 3.

**Table 2:** EBV DNA detection in MG and non-MG TETs

PATIENTS	EBV DNA	WHO histological type
MG (+) T1	+	B2
MG (+) T2	+	B2
MG (+) T3	+	B2
MG (+) T4	+	B2
MG (+) T5	+	B3
MG (+) T6	+	SCC
MG (+) T7	+	SCC
MG (+) T8	-	AB
MG (+) T9	-	B2
MG (+) T10	-	B2
MG (+) T11	-	B2
MG (-) T1	+	AB
MG (-) T2	+	AB
MG (-) T3	+	AB
MG (-) T4	+	AB
MG (-) T5	+	AB
MG (-) T6	+	AB
MG (-) T7	+	AB
MG (-) T8	+	AB
MG (-) T9	+	B1
MG (-) T10	+	B2
MG (-) T11	+	B2
MG (-) T12	+	B2
MG (-) T13	+	B2
MG (-) T14	+	B2
MG (-) T15	+	B2
MG (-) T16	+	B2
MG (-) T17	+	B2
MG (-) T18	+	B2
MG (-) T19	+	B2
MG (-) T20	+	B2
MG (-) T21	+	B3
MG (-) T22	+	B3
MG (-) T23	+	B3
MG (-) T24	+	SCC
MG (-) T25	+	SCC
MG (-) T26	+	SCC
MG (-) T27	+	SCC
MG (-) T28	+	SCC
MG (-) T29	+	SCC
MG (-) T30	+	SCC
MG (-) T31	+	SCC
MG (-) T32	+	SCC
MG (-) T33	+	SCC
MG (-) T34	+	SCC
MG (-) T35	+	SCC
MG (-) T36	-	A
MG (-) T37	-	A
MG (-) T38	-	A
MG (-) T39	-	AB
MG (-) T40	-	AB
MG (-) T41	-	AB
MG (-) T42	-	AB
MG (-) T43	-	AB
MG (-) T44	-	AB
MG (-) T45	-	AB
MG (-) T46	-	AB
MG (-) T47	-	AB
MG (-) T48	-	AB
MG (-) T49	-	AB
MG (-) T50	-	AB
MG (-) T51	-	AB
MG (-) T52	-	AB
MG (-) T53	-	AB
MG (-) T54	-	AB
MG (-) T55	-	AB
MG (-) T56	-	AB
MG (-) T57	-	B1
MG (-) T58	-	B1
MG (-) T59	-	B1
MG (-) T60	-	B1
MG (-) T61	-	B1
MG (-) T62	-	B2
MG (-) T63	-	B2
MG (-) T64	-	B2
MG (-) T65	-	B2
MG (-) T66	-	B2
MG (-) T67	-	B2
MG (-) T68	-	B2
MG (-) T69	-	B2
MG (-) T70	-	B3
MG (-) T71	-	B3
MG (-) T72	-	B3
MG (-) T73	-	B3
MG (-) T74	-	B3
MG (-) T75	-	B3
MG (-) T76	-	SCC

MG (+) T1-11: thymomas from MG patients; MG (-) T1-76: thymomas from non-MG patients

+: Detected; -: Negtive

**Table 3:** The association between EBV infection and MG

MG	EBV Negative	EBV Positive	p	Chi-square
Negative	41	35	0.2754 1.1898	
Positive	4	7		

Chi-square test

## Discussion

Over the past decades, shreds of evidence have emerged to indicate the involvement of EBV in various malignancies, such as Burkitt’s lymphoma, Hodgkin’s disease, and nasopharyngeal carcinomas. EBV is also present in tumors of similar morphology (lymphoepithelioma-like carcinomas) arising in a variety of organs, predominantly in stomach, salivary gland and thymus. As reports of EBV-positive TETs have been divergent and as different methods have been used to detect EBV, the role of EBV in the oncogenesis of thymoma is controversial.

In 1985, Leyvraz found that thymic carcinoma was associated with EBV infection, which was probably the earliest case report [13]. Thereafter, more and more cases were reported. Niehues T et al. reported a 14-year-old boy with EBV-associated thymic carcinoma [14]. Stéphan JL reported Epstein-Barr virus-positive undifferentiated thymic carcinoma in a 12-year-old white girl [15]. Matsuno Y reported Epstein-Barr virus DNA in a Japanese case of lymphoepithelioma-like thymic carcinoma [16]. Giordano S hypothesizes that EBV infection could have caused thymoma [17]. Fujii T also reported in three previous cases of EBV-associated thymic carcinoma, lymphoepithelioma-like thymic carcinoma was shown to be closely associated with EBV in their series [18]. Takeuchi H described the first case of EBV-associated thymic carcinoid tumor found by in situ hybridization (ISH) on paraffin-embedded Sect.  [19]. Recently, Zhang et al. conducted a systematic review of relevant studies published between January 1980 and December 2013 also suggesting that the prevalence of EBV in TET plays a minor role in TET pathogenesis [20].

The researchers above almost employed ISH to detect the EBV infection, and they reached an agreement that EBV was associated with thymic carcinoma but not thymoma. In our study, 72 thymomas and 15 thymic carcinomas were used to detect the viral genome at both DNA and RNA levels. We employed nested PCR to test the EBV DNA. The RNA levels were detected by Epstein–Barr-encoded RNA (EBER) ISH. The nested PCR results showed detectable EBV genome in none of type A thymoma, eight (29.6%) type AB, one (16.7%) type B1, fifteen (57.7%) type B2, four (40.0%) type B3 and fourteen (93.3%) thymic carcinomas, respectively. As the malignancy of TETs increases, EBV infection became higher gradually (p < 0.05). In 2004 WHO pulmonary, pleural and mediastinal tumors, type A, AB was classed into benign tumors, which also had a low EBV infection rate in our study. A close relationship between EBV infection and thymic carcinomas was found, which is consistent with the previous studies. However, only one type B2 thymoma showed discernible in situ signals by EBER ISH. And three cases of thymic carcinomas showed weak signals by EBER ISH. These results demonstrate that nested PCR is a sensitive method for screening the EBV genome in thymic epithelial tumors and thymic carcinomas are more often associated with the virus as previously reported.

There also existed some opposite opinions. Engel et al. analyzed 157 cases of TETs of Danish patients for EBV by applying in situ hybridization for EBER. All investigated cases were EBER negative. Therefore, they supposed that EBV does not seem to be implicated in the pathogenesis of TETs [21]. 16 western thymomas were investigated for the presence of Epstein-Barr virus (EBV) DNA sequences. The result showed none of the 16 thymomas contained evidence of the EBV genome. These results fail to demonstrate the EBV genome in western thymomas and stand in contrast to those of McGuire who previously reported that the EBV genome is present in thymomas occurring in southern Chinese patients [22].

Recently, it is reported that EBV is involved in thymoma-associated myasthenia gravis [23, 24]. Here, we also analyzed the relationship between EBV infection and thymoma-associated myasthenia gravis.However, we found no significant difference between EBV infection and thymoma-associated myasthenia gravis in this research (p = 0.2754) Table 3, perhaps we did not involve a sufficient sample size.

## Conclusion

Generally speaking, several factors influence the judge about the roles EBV played in TETs. Firstly, EBV is a widespread infection in the population, with an infection rate of more than 90% in children aged 3 to 5 years. And the infected person would carry the virus for life. Secondly, the EBV infection rate is different due to different regions and environments. This explained why EBV associated TETs were different in different parts of the world. Thirdly, EBV positive rates were inconsistent due to different sensitivity and specificity of the detection methods. More researches still need to be done to figure out the relationship between TETs and EBV.
